# Antioxidant and Fluorescence Properties of Hydrogenolyzised Polymeric Proanthocyanidins Prepared Using SO_4_^2−^/ZrO_2_ Solid Superacids Catalyst

**DOI:** 10.3390/molecules23102445

**Published:** 2018-09-25

**Authors:** Liwen Ni, Fanbin Zhao, Bolun Li, Tong Wei, Hang Guan, Shixue Ren

**Affiliations:** College of Material Science and Engineering, Northeast Forestry University, Harbin 150040, China; n3272644556@163.com (L.N.); zhaofanbinin@163.com (F.Z.); tobykyzy@163.com (B.L.); 18845780414@163.com (T.W.); 18845892378@163.com (H.G.)

**Keywords:** proanthocyanidins, SO_4_^2−^/ZrO_2_ solid superacid, catalytic hydrogenolysis, antioxidant activity, fluorescence

## Abstract

Larix bark oligomeric proanthocyanidins (LOPC) were prepared from larix bark polymeric proanthocyanidins (LPPC) by catalytic hydrogenolysis using SO_4_^2−^/ZrO_2_ solid superacid as the catalyst. The catalyst to polymeric proanthocyanidins ratio was 0.2:1 (*m*/*m*). The LOPC, obtained after hydrogenolysis at 100 °C for 4 h under 3 MPa hydrogen pressure, retained the structural characteristics of proanthocyanidins. The average degree of polymerization was reduced from 9.50% to 4.76% and the depolymerization yield was 53.85%. LOPC has good antioxidant properties and, at the same concentration, the reducing ability of LOPC was much higher than that of LPPC. The IC_50_ values of LOPC for scavenging DPPH^•^ and ABTS^•+^ radicals were 0.046 mg/mL and 0.051 mg/mL, respectively. LOPC is biocompatible and has fluorescent properties that are affected by external factors, such as solvent polarity, pH and the presence of different metal ions. These features indicate that LOPC could be developed as a new biological fluorescent marker. The depolymerization of low-value polymeric proanthocyanidins to provide high-value oligomeric proanthocyanidins and the development of new applications for proanthocyanidins represent significant advances.

## 1. Introduction

**Figure 1:**
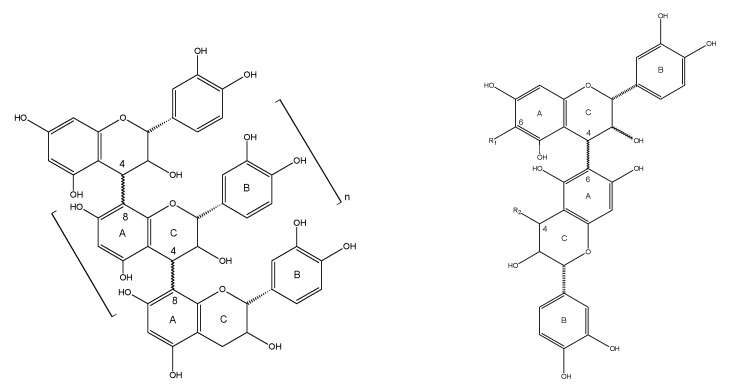
Polymeric procyanidin units containing C_4_-C_8_ bonds (**left**) and C_4_-C_6_ bonds (**right**).

Since China implemented the “Natural Forest Protection Project”, which completely bans the commercial harvesting of natural forests, the Russian Far East has exported a large amount of larch (*Larix* spp.) timber to China. After processing at a local level, this imported larch produces a mountain of wood processing residues, including bark and wood flour, which are classed as low value resources and are typically used as fuel. The bark is, however, rich in procyanidins, which account for 10–16% of the total bark mass. Procyanidins are multimeric polyphenolic compounds that are formed by connecting catechins (or epicatechins) through C_4_-C_6_ or C_4_-C_8_ bonds. The general structure is shown in Figure 1 [1,2,3,4]. Procyanidins can also be considered to be flavonoids, with a flavan-3-ol monomer structure and tannin-like properties [5].

The average degree of polymerization in larch bark polymeric proanthocyanidins (LPPC) is 9–10% and the number average molecular weight is ~2800. If the molecular weight is too large, the biological activity is low and the proanthocyanidins have limited applications. Larix bark oligomeric proanthocyanidins (LOPC), on the other hand, are < 5% polymerized and have a range of biological activities. LOPC, which are widely used in the food, medicines, cosmetics and other industries, have antioxidant and free radical scavenging activity and have the potential to treat viral and cardiovascular diseases as well as cancer [6]. Oligomeric proanthocyanidins have a large, rigid and planar, conjugated π bond structure, which confers fluorescence characteristics and also show good biocompatibility [7]. They can be used to develop new materials for biofluorescence labeling, which extends the range of applications of oligomeric procyanidins. It is, therefore, desirable to be able to depolymerize low-value polymeric proanthocyanidins into high-value oligomeric proanthocyanidins. Depolymerization of polymeric procyanidins to produce oligomeric procyanidins generally involves breaking the C_4_-C_8_ linkage between C_8_ of ring A and C_4_ of ring C in the polymeric procyanidins. In the literature [8,9], methods of cleavage of C-C bonds in natural polyphenols include oxidation and hydrogenolysis. Oxidative depolymerization involves the use of oxidants to degrade polyphenols into small molecules, even down to CO_2_ and H_2_O, under the action of catalysts, light, electricity, heat, ultrasound or microwaves. Numerous byproducts are formed and it is often difficult to obtain the required oligomeric proanthocyanidins [10]. In the hydrogenolysis reaction, it is usually the C-C bond that is broken and combined with hydrogen under the action of the catalyst. The polymeric procyanidins are thus converted into two or more oligomeric proanthocyanidins hydrogenolysis products, without destroying the basic polyphenol structural units [6,11,12]. For example, hydrogenolysis of polymeric proanthocyanidins catalyzed by Pd/C led to cleavage of the C_4_-C_8_ bonds and formation of oligomeric proanthocyanidins [4,13]. Since Pd/C catalyst is relatively expensive and has some limitations for practical applications, it is important identify new catalysts for the depolymerization of polymeric proanthocyanidins. One of these, SO_4_^2−^/ZrO_2_ solid superacid, which has both Lewis and Brønsted acid functionality at the surface, has the same catalytic efficiency as liquid acid and has been used in lignin depolymerization research [14]. Since both lignin and proanthocyanidins can be regarded as natural polyphenols, it should be possible to use SO_4_^2−^/ZrO_2_ solid superacid in the depolymerization of proanthocyanidins from larch.

In the present study, we investigated the use of SO_4_^2−^/ZrO_2_ solid superacid for depolymerization of polymeric proanthocyanidins from larch. The antioxidant and fluorescent properties of the depolymerized proanthocyanidins were then determined to identify possible applications.

## 2. Results and Analysis

### 2.1. Depolymerization of LPPC

**Figure 2:**
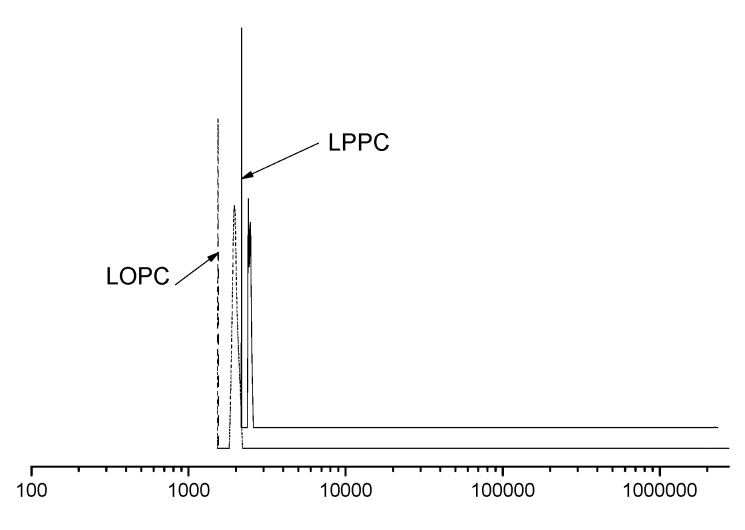
Molecular weight distribution of LPPC and LOPC.

In this study, the degree of polymerization of LPPC was 9.50%, the average degree of polymerization of LOPC was 4.76% and the depolymerization yield was 53.85%. Molecular weight measurements (Figure 2) showed that the molecular weight decreased after depolymerization.

### 2.2. Detection and Analysis of LPPC and LOPC Structures

**Figure 3:**
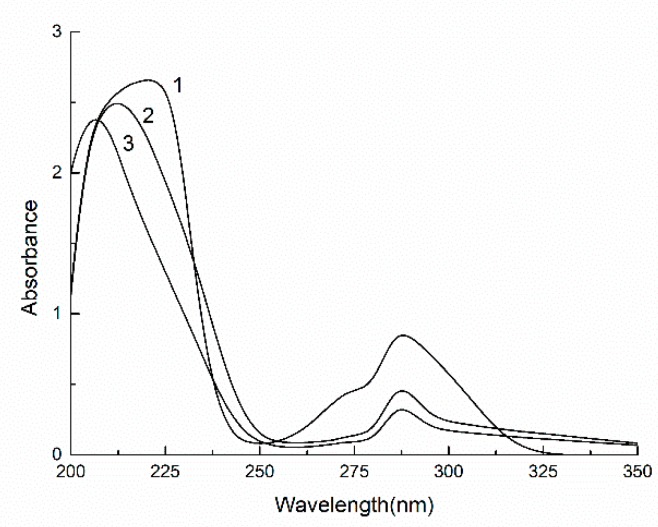
UV spectra of catechin standard (1), LOPC (2) and LPPC (3).

**Figure 4:**
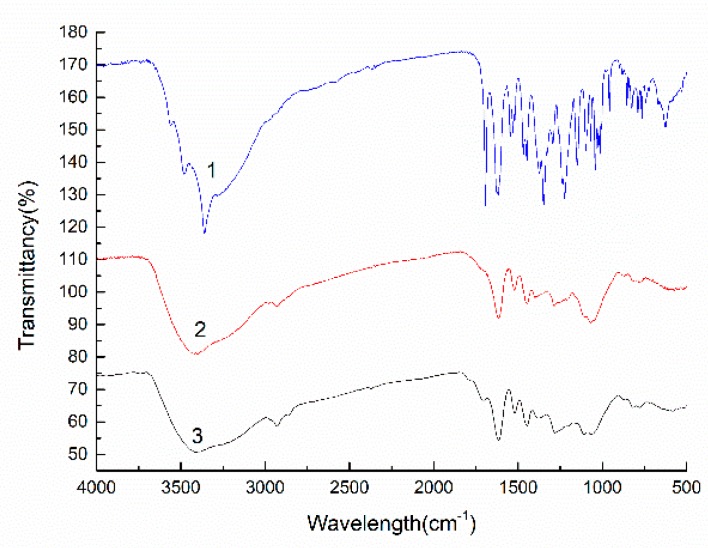
IR spectra of catechin standard (1), LOPC (2) and LPPC (3).

UV and IR spectra were used to detect LPPC and the depolymerized product LOPC (Figure 3 and Figure 4).

The UV spectrum of LOPC has a maximum absorption peak at ~298 nm, which is close to the positions of the maximum absorption peaks of the catechin standard and LPPC (Figure 3), indicating that the unit structure of LPPC has not been destroyed. The IR spectra of the catechin standard, LPPC and LOPC show a broad and strong telescopic absorption peak attributable to phenolic hydroxyl (-OH) groups at 3390 cm^−1^ (Figure 4). The elastic vibration absorption peak of methylene (C-H) groups appears at 2930 cm^−1^. Peaks at 1611 cm^−1^, 1515cm^−1^ and 1450cm^−1^ are the characteristic absorption peaks of benzene rings, the peak at 1290 cm^−1^ is the absorption peak of ether (C-O-C) stretching vibrations and the peak at 1112 cm^−1^ is characteristic of secondary alcohols. These spectral data show that SO_4_^2−^/ZrO_2_ solid superacid degrades LPPC mainly by breaking the C_4_-C_8_ bond of the polymeric proanthocyanidin units but does not destroy the molecular structure of proanthocyanidin monomers since the product LOPC retains the structural characteristics of proanthocyanidins. 

### 2.3. Antioxidant Properties of LOPC

#### 2.3.1. Reducing Power

**Figure 5:**
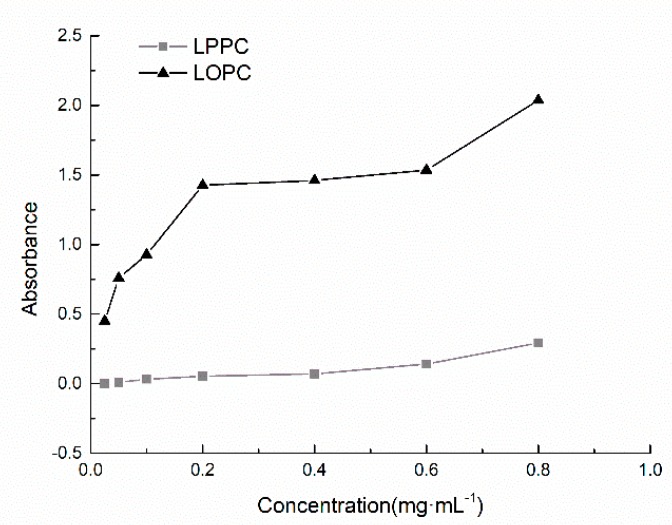
Comparison of reducing power of proanthocyanidins with different mass concentrations.

Reducing power is an important index of antioxidant activity. The stronger the reducing power, the greater the resistance to oxidation. Here, the reducing abilities of LPPC and LOPC were measured using the Prussian Blue method. The underlying principle of this method is that the phenolic hydroxyl groups on proanthocyanidin molecules can reduce Fe^3+^ to Fe^2+^ and the reducing ability of proanthocyanidin is reflected in the changes in Fe^3+^ concentration. Over the concentration range 0.025–0.8 mg/mL, the reducing power of both LPPC and LOPC increased with increasing mass concentration (Figure 5). At the same concentration, however, the reducing ability of LOPC is much greater than that of LPPC. The main reason for the enhanced reducing activity of LOPC is the increased number of phenolic hydroxyl groups. When LPPC is degraded by SO_4_^2−^/ZrO_2_ solid superacid, C_4_-C_8_ bonds in the molecule are broken and the number of phenolic hydroxyls in LOPC is thus higher than the number in LPPC.

#### 2.3.2. DPPH^•^ Scavenging Activity

**Figure 6:**
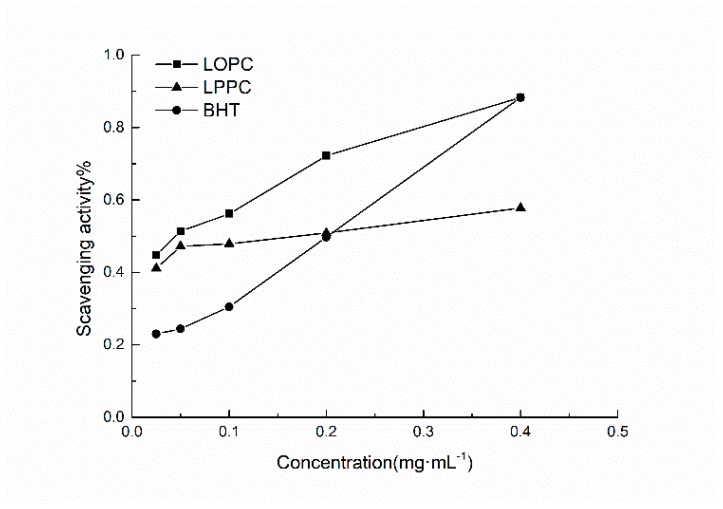
DPPH^•^ free radical scavenging ability of BHT, LPPC and LOPC with different mass concentrations.

**Figure 7:**
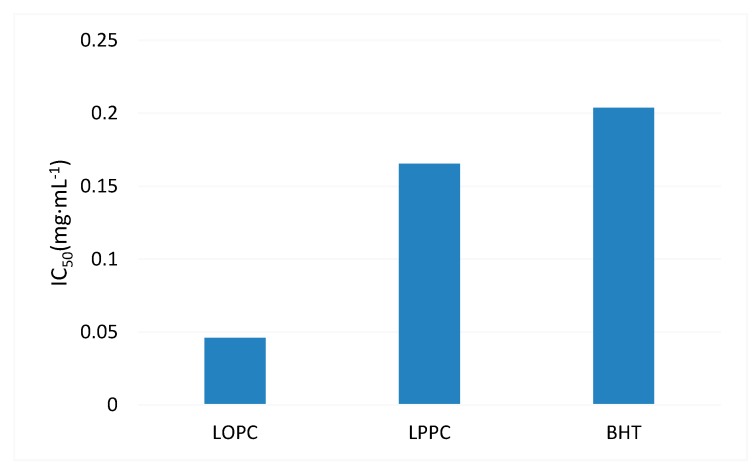
Half-maximal DPPH^•^ free radical scavenging rate (IC_50_) of BHT, LPPC and LOPC.

DPPH^•^ is a stable nitrogen-centered free radical. Its stability arises mainly as a result of its conjugated structure and the steric hindrance of the three benzene rings, which make the unpaired electrons on the intermediate nitrogen atom difficult to pair. When an antioxidant is added, self-reduction releases H^+^, which will combine with DPPH^•^. The DPPH^•^ scavenging ability of antioxidants can, therefore, be determined by the change in degree of absorbance. From a structural point of view (see Figure 1), the *o*-phenolic group of the proanthocyanidin B ring can act as a hydrogen donor to receive free radicals and free radicals generated by itself form stable intramolecular hydrogen bonds with semi-quinoid free radicals, *o*-quinones and the like, thus blocking the free radical chain reaction [6]. Obviously, proanthocyanidins also have the ability to scavenge DPPH^•^. The DPPH^•^ free radical scavenging ability of different mass concentrations of BHT (control), LPPC and LOPC are shown in Figure 6. Over the concentration range 0.025–0.4 mg/mL, the DPPH^•^ scavenging ability of LPPC, LOPC and BHT increased with increasing concentration. At the same concentration, the order of DPPH^•^ scavenging ability was LOPC > LPPC > BHT. Half-maximal scavenging rates (IC_50_) is the required mass concentration of each sample when the scavenging activity reaches 50 %. DPPH^•^ half-maximal scavenging rates (IC_50_) were also in the order LOPC > LPPC > BHT (Figure 7). Generally speaking, the smaller the IC_50_ value, the stronger the antioxidant free radical scavenging ability and the better the antioxidant performance. When LPPC is degraded by SO_4_^2−^/ZrO_2_ solid superacid, LPPC is depolymerized into LOPC and its antioxidant capacity is enhanced.

#### 2.3.3. ABTS^•+^ Scavenging Activity

**Figure 8:**
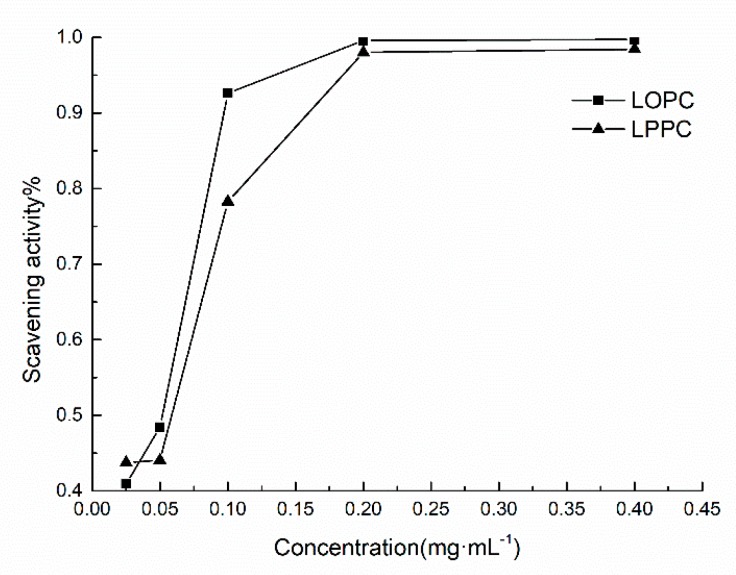
ABTS^•+^ free radical scavenging ability of LPPC and LOPC with different mass concentrations.

**Figure 9:**
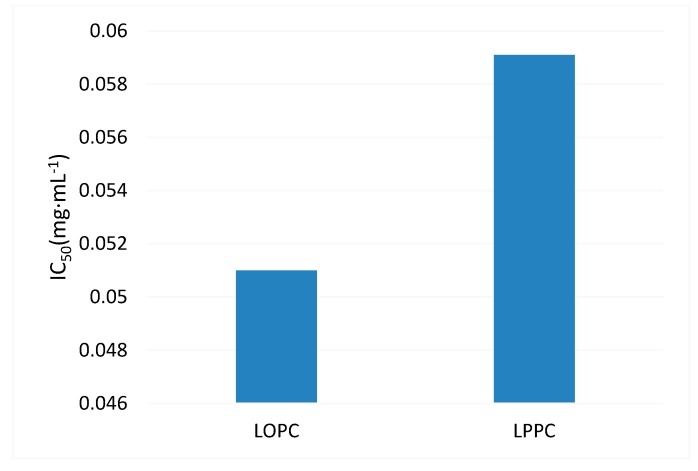
Half-maximal ABTS^•+^ free radical scavenging rate (IC_50_) of LPPC and LOPC.

ABTS^•+^ scavenging capacity is commonly used to indicate the total antioxidant capacity of antioxidants. The principle underlying this assay is that oxidants convert ABTS to green ABTS^•+^. The addition of antioxidants will inhibit the production of ABTS^•+^, resulting in a lightening of the color of the solution and a decrease in absorbance. The greater the color change of the solution, the greater the removal rate of ABTS^•+^ and the stronger the resistance to oxidation. The ability of LPPC and LOPC to scavenge ABTS^•+^ free radicals is shown in Figure 8. Over the concentration range 0.025–0.4 mg/mL, the extent of ABTS^•+^ scavenging increased with increasing concentration of LPPC and LOPC and the extent of ABTS^•+^ scavenging approached 100%. Half-maximal scavenging rates (IC_50_) show that LOPC has a greater ability to scavenge ABTS^•+^ than LPPC (Figure 9). When LPPC is depolymerized by SO_4_^2−^/ZrO_2_ solid superacid, the product LOPC better inhibits the production of ABTS^•+^ and has greater antioxidant activity than LPPC.

### 2.4. Fluorescence Characteristics of LOPC

Structurally, LOPC consists of a large, rigid and planar, conjugated π bond structure, which has fluorescent properties. Here, we have investigated the influence of external factors, such as solvent polarity, pH and the presence of different metal ions, on the fluorescence properties of LOPC.

#### 2.4.1. Effect of Solvent Polarity on Fluorescence Characteristics of LOPC

**Figure 10:**
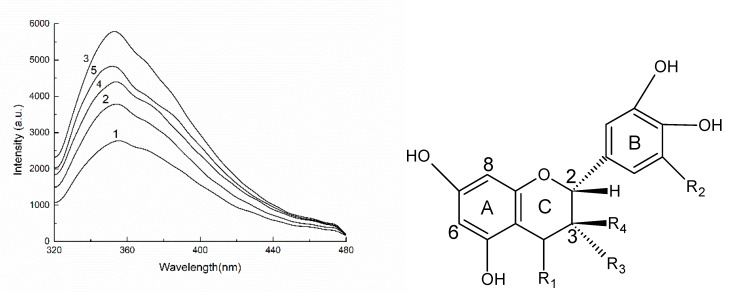
Effect of solvent polarity on fluorescence intensity of LOPC. Ethanol concentration: 1, 5%; 2, 25%; 3, 50%; 4, 75%; 5, 100%.

By comparing the fluorescence intensity of LOPC under excitation with light of different wavelengths, the optimal wavelength (λ_ex_) of excitation light was determined to be 320 nm (Section 3.5.1). We now prepared LOPC solutions (0.1 mg/mL) in aqueous ethanol with 100%, 75%, 50%, 25% and 5% ethanol (*v*/*v*) to determine the effect of increasing the polarity of the solvent. The fluorescence intensity was measured at an excitation wavelength (λ_ex_) of 320 nm. As shown in Figure 10, solvent polarity affects the position of the maximum fluorescence emission peak of LOPC. When the ethanol concentrations were 100%, 75%, 50%, 25% and 5%, the maximum fluorescence emission peak positions (E_m_) were 352, 354, 353, 354 and 355 nm, respectively. The fluorescence spectrum was thus redshifted as the proportion of ethanol decreased but the displacement range was not large, only 1–3 nm. It is generally believed that the shift of the fluorescence spectrum to longer wavelength can mostly be attributed to π→π* transitions and intramolecular charge transfer transitions, accompanied by rearrangement of electrons, which results in larger changes in dipole momen. The polarity of the solvent also affects the fluorescence intensity of the LOPC, the fluorescence intensity of the LOPC solutions of different ethanol concentrations: 50% > 100% > 75% > 25% > 5%, the reason is that the LOPC molecule contains a large number of phenolic hydroxyl and the formation of hydrogen bonds between the phenolic hydroxyl groups and H_2_O in the solvent increases the fluorescence intensity. However, when the solvent polarity is too large, the LOPC fluorescence intensity will be reduced. Therefore, it is advisable to increase the polarity of the solvent to increase the fluorescence intensity of LOPC.

#### 2.4.2. Effect of pH on Fluorescence Characteristics of LOPC

**Figure 11:**
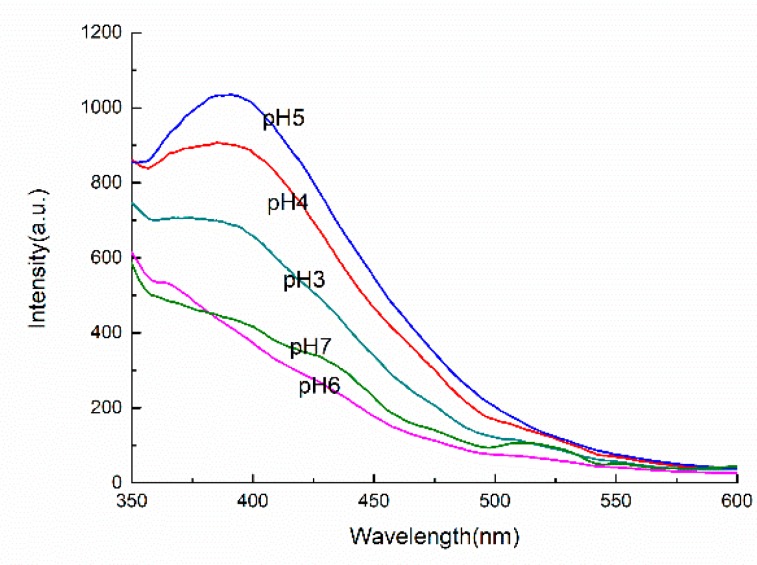
Effect of pH on fluorescence intensity of LOPC.

LOPC contains acidic phenolic hydroxyl groups. Under different pH conditions, the dissociation of the phenolic hydroxyl groups alters, which may change the nature and rate of non-radiative transition processes. Since non-radiative transitions compete with the luminescence process, the fluorescence spectrum and the intensity of emission of LOPC is expected to vary with pH. Since the polarity of the solvent also affects fluorescence intensity, we investigated the fluorescence spectra of LOPC (0.1 mg/mL) in ethanol solution under different pH conditions (Figure 11).

When 3 ≤ pH ≤ 5, the LOPC fluorescence spectrum had the largest emission peak and the position of the fluorescence peak was ~390 nm. At pH > 5, the fluorescence peak disappeared. The phenolic groups of LOPC act as electron donors. At pH ≤ 5, protons will dissociate, leading to a change in the energy gap between the ground state and the excited state of the LOPC molecule and shifting the luminescence spectrum.

The pH of the solution also affects the fluorescence intensity of LOPC. When analyzing the fluorescence of LOPC, the analysis should be carried out at pH 5, which will improve the sensitivity and accuracy of fluorescence measurements.

#### 2.4.3. Effects of Different Metal Ions on Fluorescence Properties of LOPC

**Figure 12:**
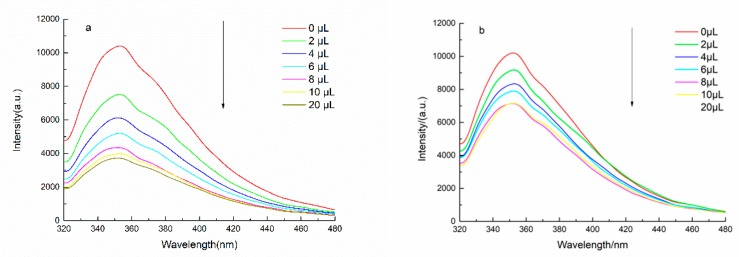

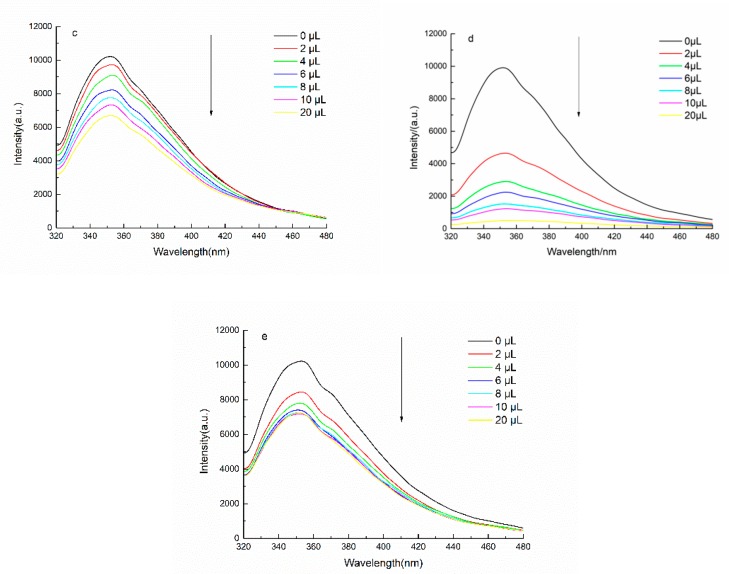
Effect of (**a**) Cu^2+^, (**b**) Ba^2+^, (**c**) Al^3+^, (**d**) Fe^3+^ and (**e**) Ni^2+^ ions on fluorescence intensity of LOPC.

In order to investigate the effect of metal ions on the fluorescence properties of LOPC, salt solutions (10 mg/mL) containing Cu^2+^, Ba^2+^, Al^3+^, Fe^3+^ and Ni^2+^ ions were added to a solution (0.1 mg/mL) of LOPC in 50% (*v*/*v*) aqueous ethanol. The relationship between the concentration of metal ions and the fluorescence intensity of LOPC was studied at an excitation wavelength of 320 nm (Figure 12). The fluorescence intensity of LOPC gradually decreased with increasing concentration of metal ions. The decrease in fluorescence intensity is mainly because Cu^2+^, Ba^2+^, Al^3+^, Fe^3+^ and Ni^2+^ ions form complexes with the phenolic hydroxyl groups in LOPC. As the concentration of metal ions increased, the concentration of LOPC in the solution decreased, resulting in a decrease in the fluorescence intensity of LOPC.

**Figure 13:**
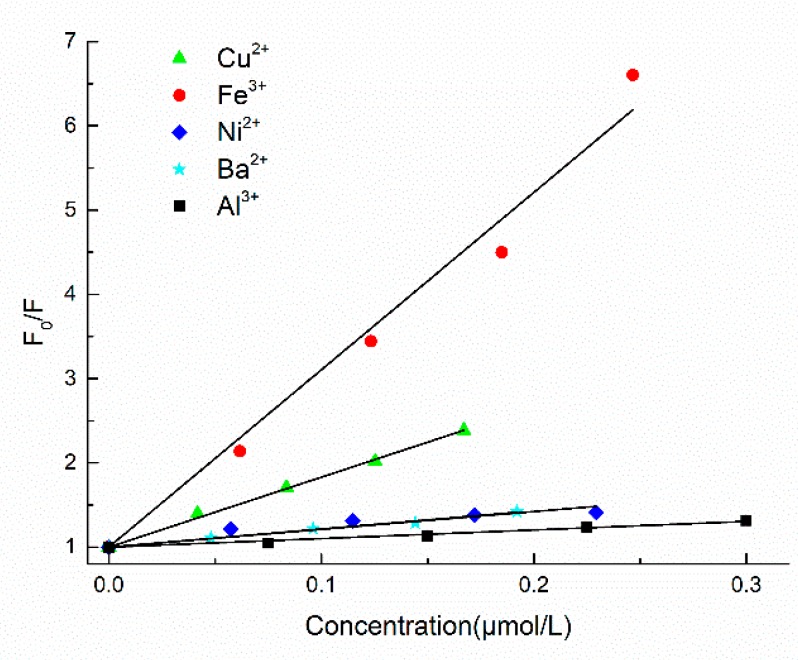
Stern-Volmer curves for quenching of LOPC fluorescence by Cu^2+^, Ba^2+^, Al^3+^, Fe^3+^ and Ni^2+^ ions.

A curve showing the relationship between the degree of fluorescence quenching (F_0_/F) of LOPC and the concentrations of Cu^2+^, Ba^2+^, Al^3+^, Fe^3+^ and Ni^2+^ ions can be obtained using the Stern-Volmer equation [15] (Figure 13). The complexation constants of different metals can be calculated by fitting to the Stern-Volmer curves (Table 1).

Because of the diverse structural elements of LOPC and the existence of intermolecular interaction forces and space-resistance effects, metal ions have different abilities to form complexes with phenolic groups in the structural unit of LOPC. The quenching effects of different metal ions are also different.

By comparing complexation constants, the abilities of metal ions to form complexes with LOPC were determined to be: Fe^3+^ > Cu^2+^ > Ba^2+^ > Ni^2+^ > Al^3+^. According to the theory of soft and hard acids (SHAB) [16,17], the central metal ion in the complex is acidic and the ligand is basic. Al^3+^ and Fe^3+^ are hard acids, Cu^2+^ and Ni^2+^ are borderline acids and Ba^2+^ is a soft acid, whilst the polyphenol compound, LOPC, shows alkalinity [15]. This means that, theoretically, Al^3+^ and Fe^3+^ are more likely to form metal ion complexes with LOPC. The experimental results show, however, that Cu^2+^ and Fe^3+^ have greater ability to form complexes with LOPC than Ba^2+^, Ni^2+^ and Al^3+^. The reason is for this apparent discrepancy is that, normally, phenolic groups that are not involved in the dehydrogenation reaction are unlikely to react with metal ions but, under certain pH conditions, phenolic groups are dehydrogenated, generating charged oxygen atoms that form complexes with acids. In the presence of Fe^3+^, Cu^2+^ or other specific metal ions, LOPC is easily dehydrogenated at pH 5–8 and undergoes complex reactions. Al^3+^ is easily hydrolyzed and the concentration of Al^3+^ in AlCl_3_ solution (10 mg/mL) is 0.0414 M. According to Equation (1)

(1) $$\mathrm{pH}=3.03-\frac{1}{3}\mathrm{lg}\left[{\mathrm{Al}}^{3+}\right]$$

Al^3+^ ions begin to hydrolyze when the pH is 3.49. The pH of the LOPC solution is 5.25, which indicates that Al^3+^ is hydrolyzed to Al(OH)_3_ in the reaction system. The decrease in Al^3+^ concentration leads to a decrease of the complexation constant and reduced ability to form complexes with LOPC.

## 3. Materials and methods

### 3.1. Experimental Materials, Reagents and Instruments

Crushed larch bark was obtained from Inner Mongolia. 2,2-Diphenyl-1-(2,4,6-trinitrophenyl)hydrazyl (DPPH), 2,2′-azino-bis(3-ethylbenzothiazoline-6-sulfonic acid) diammonium salt (ABTS) and 2,6-di-tert-butyl-4-methylphenol (BHT) were all biological grade; other reagents were analytical grade. Equipment used in the study included a Zolix combination fluorescence spectrometer (Beijing Zhuoli Hanguang Instrument Co., Ltd., Beijing, China), an El20 laboratory pH meter (Mettler Toledo Instruments Shanghai Co., Ltd., Shanghai, China), a TU-1950 dual beam ultraviolet visible spectrophotometer (Beijing Puxi General Instrument Co., Ltd., Beijing, China), a vacuum oven (Shanghai Yiheng Scientific Instrument Co., Ltd., Shanghai, China), a GCF-1 autoclave controller (Zhiyun Automation Equipment Co., Ltd., Dalien, China), RE-52AA rotary evaporator (Shanghai Yarong Biochemical Instrument Factory, Shanghai, China), a 2XZ (S)-4 rotary vane vacuum pump (Shanghai Deying Vacuum and Lighting Equipment Co., Ltd., Shanghai, China) an SX2-4-10 box resistance furnace (Tianjin Tianyu Experimental Instrument Co., Ltd., Tianjin, China) and an FTIR-650 Fourier transform infrared spectrometer (Tianjin Gangdong Science and Technology Development Co., Ltd., Tianjin, China).

### 3.2. Sample Preparation

#### 3.2.1. Preparation of LPPC

Crushed larch bark (80 g, particle size 0.5–1.0 mm) was refluxed with 70% (*v*/*v*) ethanol-water (800 mL) for 120 min and then filtered. The filter cake was refluxed for 60 min with 70% (*v*/*v*) ethanol-water (540 mL) and then filtered. The combined filtrates, which had a total volume of ~1200 mL, were extracted with an equal volume of petroleum ether to remove impurities such as resin and gum. The purified filtrate was evaporated under reduced pressure using a rotary evaporator (RE-52B, Shanghai Yarong Biochemical Instrument Factory, Shanghai, China; bath temperature 45 ± 5 °C) to remove ethanol, leaving a volume of ~300 mL. Insoluble red material was removed by filtration and the resulting concentrated solution was extracted 6 times with an equal volume of ethyl acetate, when the ethyl acetate layer was colorless. The aqueous solution was concentrated under reduced pressure to ~50 mL (bath temperature 55 ± 5 °C) and the concentrated solution was dried under vacuum at 50 ± 2 °C to provide LPPC (3.86 g).

#### 3.2.2. Preparation of SO_4_^2−^/ZrO_2_ Solid Superacid Catalyst

Powdered zirconium hydroxide (5 g) was dissolved in 1 M sulfuric acid (100 mL). The solution was allowed to stand for 12 h and the resulting solid was removed by filtration. The filter cake was dried in a vacuum oven at 105 °C for 12 h and then placed in a furnace-resistant crucible and calcined in a box resistance furnace at 550 °C for 3.5 h to provide SO_4_^2−^/ZrO_2_ solid superacid catalyst. 

#### 3.2.3. Depolymerization of LPPC

SO_4_^2−^/ZrO_2_ solid superacid (0.35 g) was added to a solution of LPPC (1.5 g, labeled m_1_) in 70% (*v*/*v*) ethanol-water (100 mL). The solution containing LPPC and catalyst was transferred to a high-pressure reaction kettle and the vessel was purged with nitrogen. The vessel was then charged with hydrogen to a pressure of 3 MPa. The air inlet valve was sealed and the contents of the vessel were stirred at 500 r/min and 100 ± 2 °C for 4 h. The product-containing solution was concentrated under reduced pressure (bath temperature 35 ± 5 °C) to ~30 mL and then extracted with equal volumes of ethyl acetate until colorless. The aqueous solution was concentrated under reduced pressure (bath temperature 35 ± 5 °C) and then dried under vacuum at 50 ± 2 °C to provide LOPC (0.8078 g, labeled m_2_). 

### 3.3. Characterization of Proanthocyanidins

#### 3.3.1. Determination of Average Degree of Polymerization and Degradation Rate

The average degree of polymerization of proanthocyanidins was determined using the vanillin-hydrochloric acid method [18].

(2) $$\mathrm{Average}\;\mathrm{degree}\;\mathrm{of}\;\mathrm{polymerization}\;D_{n}=\frac{\rho_{n}\times 1000}{M\times c}$$

In Equation (2), ρ_n_ is the mass concentration (μg/mL), M is the molar mass (g/mol) and c is the molar concentration (mmol/mL).

The degradation yield can be calculated using formula (3).

(3) $$\mathrm{Degradation}\;\mathrm{yield}\;(\%)=\frac{m_{2}}{m_{1}}\times 100\%$$

In Equation (3), m_1_ is the quality (1.5 g) of polymeric proanthocyanidins before depolymerization (g) and m_2_ is the quality (0.8078 g) of oligomeric proanthocyanidins after depolymerization (g).

#### 3.3.2. Molecular Weight Distribution

Two sample of LPPC (0.0200 g) and LOPC (0.0203 g) were dissolved in 70% (*v*/*v*) ethanol-water (10 mL) and the solution was filtered through a 0.45 μm membrane. The molecular weight distribution of proanthocyanidins was determined using an Agilent 1100 HPLC system (Agilent Technologies, Santa Clara, CA, USA), equipped with tandem gel columns, 79911GF-084 and 79911GF-083)). The injection volume was 50 μL and the column temperature was 30 °C. A diode array detector (Agilent Technologies, Santa Clara, CA, USA) was used, with a detection wavelength of 270 nm. The mobile phase was 70% (*v*/*v*) ethanol-water and the flow rate was 1.0 mL/min.

#### 3.3.3. Spectral Analysis

For infrared spectroscopy, the sample was prepared as a KBr disc. Sample (2 mg) was ground with spectroscopic grade KBr powder (100 mg). The sample was tableted and then scanned using an infrared spectrometer (Tianjin Gangdong Science and Technology Development Co., Ltd., Tianjin, China) over the range 4000–500 cm^−1^, with resolution of 2 cm^−1^. Sixteen scans were collected [19]. For UV spectroscopy, the sample was dissolved in ethanol at a concentration of 40 μg/mL. The solution was scanned over the range 200–700 nm using a UV-Vis spectrometer (Beijing Puxi General Instrument Co., Ltd., Beijing, China).

### 3.4. Determination of Antioxidant Activity of Proanthocyanidins

The reducing ability of LOPC was tested using the Prussian Blue method [20]. The degradation products LOPC were prepared into solutions with concentrations of 0.025, 0.05, 0.1, 0.2, 0.4, 0.6 and 0.8 g/L, respectively. 1 mL of LOPC solution with different concentrations were respectively transferred to test tubes and 2.5 mL of phosphate buffer solution (pH 6.6) and 2.5 mL of potassium ferricyanide solution (mass fraction 1%) were added. After thorough mixing, the mixture was kept at 50 °C for 20 min, 2.5 mL of trichloroacetic acid was added to shake well, 2.5 mL of mixed solution was transferred, 2.5 mL of distilled water and 0.5 mL of FeCl_3_ (mass fraction 0.1%) were added to shake well and absorbance was measured at 700 nm wavelength by TU-1950 double-beam UV-Vis spectrometer (Beijing Puxi General Instrument Co., Ltd., Beijing, China). The same method was used to measure LPPC’s restoring ability and compare it with LOPC’s.

DPPH^•^ free radical scavenging capacity [21] was determined colorimetrically. The DPPH solution with the concentration of 0.04 mg/mL was prepared with absolute ethanol as solvent and stored at 0–4 °C in the dark. 1 mL of LOPC solution with different concentrations were respectively transferred to test tubes and a proper amount of DPPH solution was added. After thorough mixing, the mixture was left in the dark at room temperature for 30 min and the absorbance was measured at 517 nm wavelength, which was designated A_1_. The absorbance of LOPC solution with a concentration of 0 is recorded as A_0_. BHT was used as the positive control of LOPC. The DPPH radical scavenging ability of LPPC was compared with that of LOPC by the same method.

$$\mathrm{Calculation}\;\mathrm{formula}:\;\mathrm{Scavenging}\;\mathrm{ability}\;(\%)=\frac{A_{0}-A_{1}}{A_{0}}\times 100\%\;$$

ABTS^•+^ free radical cation scavenging capacity [22] was also determined colorimetrically. Using 2.45 mmol/µL potassium persulfate solution as solvent to prepare BTS stock solution with concentration of 7 mmol/µL, dilute ABTS stock solution with 10 mmol/µL phosphoric acid buffer solution (pH 7.4) until its absorbance reaches 0.7 ± 0.02 at 734 nm wavelength, thus obtaining ABTS determination solution. 4 µL of LOPC solution with different concentrations were transferred to a test tube and 4 mL of ABTS measurement solution was added. After thorough mixing, the solution was allowed to stand for a period of time and the absorbance was measured at 734 nm wavelength, which was recorded as a sample A.

$$\mathrm{Calculation}\;\mathrm{formula}:\;{\mathrm{ABTS}}^{•+}\mathrm{scavenging}\;\mathrm{ability}\;(\%)=\left(1-\frac{A_{\mathrm{sample}}}{0.700}\right)\times 100\%\;$$

The ABTS^•+^ free radical scavenging ability of LPPC was measured by the same method and compared with that of LOPC.

### 3.5. Determination of Fluorescent Properties of Proanthocyanidins

#### 3.5.1. Determination of Optimal Wavelength of Excitation Light

LOPC was dissolved in 50% (*v*/*v*) ethanol-water to prepare a solution with concentration 0.1 g/L. An aliquot (3000 µL) of the solution was placed in a quartz cell and the emission spectrum was recorded over the range 280–600 nm, with excitation at 280, 300, 320, 340 and 360 nm. The optimal excitation wavelength (λ_ex_) was determined by measuring the intensity of the emission spectra. 

#### 3.5.2. Effects of Different Metal Ions on Fluorescent Properties of Degraded Proanthocyanidins

Degraded proanthocyanidins were dissolved in 50% (*v*/*v*) ethanol-water to prepare a solution of LOPC with a concentration of 0.1 mg/mL. The pH value of the LOPC solution was determined to be 5.25 using a pH meter (Mettler Toledo Instruments Shanghai Co., Ltd., Shanghai, China). Aliquots (3000 µL) of the degraded proanthocyanidin solution were placed in quartz cells at room temperature and treated with different amounts (2, 4, 6, 8, 10 and 20 µL) of aqueous solutions of CuSO_4_, BaCl_2_, FeCl_3_, AlCl_3_ and Ni(NO_3_)_2_ (all 10 mg/mL). After stirring at 1500 r/min for 5 min, the emission spectra of the metal salt-containing solutions were recorded with an excitation wavelength of 320 nm (λ_ex_).

#### 3.5.3. Fluorescent Properties of LOPC

##### 3.5.3.1 Effect of Solvent Polarity on Fluorescent Properties of LOPC

Samples of LOPC (1.0 mg) were dissolved in ethanol-water mixtures with different volume ratios to prepare LOPC solutions with a volume of 10 mL and a concentration of 0.1 mg/mL. The ethanol concentrations were 5%, 25%, 50%, 75% and 100%. Aliquots (3000 µL) of the LOPC solutions were pipetted into quartz cells and the emission spectra were recorded over the range 320–600 nm, with an excitation wavelength of 320 nm (λ_ex_).

##### 3.5.3.2 Effect of pH on Fluorescent Properties of LOPC

Citric acid/sodium citrate buffer solutions (50 mM) with pH values of 3, 4 and 5 and sodium dihydrogen phosphate/disodium hydrogen phosphate buffer solutions (50 mM) with pH values of 6 and 7 were prepared. A pH meter was used to accurately measure the pH of the buffer solutions. Aliquots (2900 µL) of buffer solutions with different pH values (3, 4, 5, 6 and 7) were pipetted into 1 cm quartz cells and aliquots of LOPC solution in absolute ethanol (0.1 mg/mL, 100 µL) were added. At this point, the LOPC concentration was ~3.3 × 10^−3^ mg/mL and the ethanol concentration was 3.3%. After the solution was well mixed, the emission spectrum was measured over the range 320–600 nm, with an excitation wavelength of 320 nm (λ_ex_).

## 4. Conclusions

In the present study, we have investigated the catalytic hydrogenolysis of proanthocyanidins from larch bark using SO_4_^2−^/ZrO_2_ solid superacid as the catalyst. We have also measured the antioxidant and fluorescent properties of the oligomeric procyanidins after hydrogenolysis. The main conclusions of the study are as follows:

(1). SO_4_^2−^/ZrO_2_ solid superacid catalyzes hydrogenolysis of proanthocyanidins from larch bark. The hydrogenolysis conditions were: SO_4_^2−^/ZrO_2_ solid superacid: polyproanthocyanidin mass ratio, 0.2:1; reaction temperature, 100 °C; reaction time, 4 h; hydrogen pressure, 3 MPa. Using these conditions, the average degree of polymerization was reduced from 9.50% to 4.76% and the depolymerization yield was 53.85%. The LOPC obtained using this process retained the structural characteristics of proanthocyanidins. 

(2). The depolymerization product, LOPC, has good antioxidant properties. At the same concentration, the reducing ability of LOPC was much higher than that of LPPC. The IC_50_ values of LPPC for scavenging DPPH^•^ and ABTS^•+^ were 0.046 mg/mL and 0.051 mg/mL, respectively.

(3). The depolymerization product LOPC, which has a large, rigid and planar, conjugated π bond structure, has fluorescence properties. External factors, such as solvent polarity, pH and the presence of different metal ions, have an effect on the nature and intensity of the fluorescence emission of LOPC. As the polarity of the solvent increases, the maximum emission peak of LOPC shifts to longer wavelength. pH also affects the intensity of the fluorescence emission of LOPC; when the pH of the solution is 5, the fluorescence intensity of LOPC is high. Metal ions can quench the fluorescence of LOPC but the chemical structure of LOPC remains unchanged.

The depolymerization of low-value polymeric proanthocyanidins to provide high-value oligomeric proanthocyanidins and the development of new applications for proanthocyanidins represent significant advances. Since LOPC is biocompatible and has fluorescence characteristics that are affected by changes in the environment, it has the potential to be developed as a novel fluorescent labeling material for biological studies.

## Figures and Tables

**Table 1:** Complexation constants (K_sv_) for complexation between LOPC and metal ions.

Complex	Metal Ion Concentration (μmol/L)	Maximum Emission Wavelength of Complex (nm)	Complexation Constant Ksv (× 10^6^ L/mol)	$R^{2}$	$\sqrt{s^{2}}$
Al^3+^-PC	≤ 0.15	350	1.02	0.99967	0.00174
Cu^2+^-PC	≤ 0.15	350	8.31	0.99973	0.00335
Ba^2+^-PC	≤ 0.15	350	2.16	0.99987	0.00076
Fe^3+^-PC	≤ 0.15	350	21.05	0.99422	0.37551
Ni^2+^-PC	≤ 0.15	350	2.115	0.99694	0.01984
